# Therapeutic effect of combination vitamin D3 and siponimod on remyelination and modulate microglia activation in cuprizone mouse model of multiple sclerosis

**DOI:** 10.3389/fnbeh.2022.1068736

**Published:** 2023-01-05

**Authors:** Kholoud M. Al-Otaibi, Badrah S. Alghamdi, Maryam A. Al-Ghamdi, Rasha A. Mansouri, Ghulam Md Ashraf, Ulfat M. Omar

**Affiliations:** ^1^Department of Biochemistry, Faculty of Science, King Abdulaziz University, Jeddah, Saudi Arabia; ^2^Department of Chemistry, Faculty of Science, Albaha University, Albaha, Saudi Arabia; ^3^Department of Physiology, Neuroscience Unit, Faculty of Medicine, King Abdulaziz University, Jeddah, Saudi Arabia; ^4^Pre-Clinical Research Unit, King Fahd Medical Research Center, King Abdulaziz University, Jeddah, Saudi Arabia; ^5^Vitamin D Pharmacogenomics Research Group, King Abdulaziz University, Jeddah, Saudi Arabia; ^6^Experimental Biochemistry Unit, King Fahd Medical Research Center, King Abdulaziz University, Jeddah, Saudi Arabia; ^7^Department of Medical Laboratory Sciences, Faculty of Applied Medical Sciences, King Abdulaziz University, Jeddah, Saudi Arabia; ^8^Princess Dr. Najla Bint Saud Al-Saud Center for Excellence Research in Biotechnology, King Abdulaziz University, Jeddah, Saudi Arabia

**Keywords:** vitamin D3, siponimod, cuprizone, multiple scleorsis, remyelination, microglia markers

## Abstract

Stimulation of remyelination is critical for the treatment of multiple sclerosis (MS) to alleviate symptoms and protect the myelin sheath from further damage. The current study aimed to investigate the possible therapeutic effects of combining vitamin D3 (Vit D3) and siponimod (Sipo) on enhancing remyelination and modulating microglia phenotypes in the cuprizone (CPZ) demyelination mouse model. The study was divided into two stages; demyelination (first 5 weeks) and remyelination (last 4 weeks). In the first 5 weeks, 85 mice were randomly divided into two groups, control (*n* = 20, standard rodent chow) and CPZ (*n* = 65, 0.3% CPZ mixed with chow for 6 weeks, followed by 3 weeks of standard rodent chow). At week 5, the CPZ group was re-divided into four groups (*n* = 14) for remyelination stages; untreated CPZ (0.2 ml of CMC orally), CPZ+Vit D3 (800 IU/kg Vit D3 orally), CPZ+Sipo (1.5 mg/kg Sipo orally), and CPZ+Vit D3 (800 IU/kg Vit D3) + Sipo (1.5 mg/kg Sipo orally). Various behavioral tasks were performed to evaluate motor performance. Luxol Fast Blue (LFB) staining, the expression level of myelin basic protein (MBP), and M1/M2 microglia phenotype genes were assessed in the corpus callosum (CC). The results showed that the combination of Vit D3 and Sipo improved behavioral deficits, significantly promoted remyelination, and modulated expression levels of microglia phenotype genes in the CC at early and late remyelination stages. These results demonstrate for the first time that a combination of Vit D3 and Sipo can improve the remyelination process in the cuprizone (CPZ) mouse model by attenuating the M1 microglia phenotype. This may help to improve the treatment of MS patients.

## 1. Introduction

Multiple sclerosis (MS) is a chronic, demyelinating, progressive disease of the central nervous system (CNS) characterized by inflammation, neurodegeneration, and axonal losses, accompanied by highly variable clinical complications and histopathological manifestations (Ghasemi et al., 2017; Thompson and Tsirka, 2020). It is a common neurological disorder worldwide and the most common cause of nontraumatic neurological disability among young adults in several countries. The disease affects more than 2.5 million people worldwide and imposes a significant health, economic, and psychological burden on patients and society (Browne et al., 2014).

Previous studies have shown that activated microglia and astrocytes play a pivotal role in disease onset and progression by participating in the inhibition or development of demyelination in MS (Freeman et al., 2017; Petković et al., 2017; Thompson and Tsirka, 2020). Microglia have distinct phenotypes: the classical activated phenotype (M1; pro-inflammatory) and the alternative phenotype (M2; anti-inflammatory), each producing a wide range of mediators and having their own physiological functions. M1 microglia releases pro-inflammatory cytokines such as interleukin 1β (IL-1β), tumor necrosis factor-alpha (TNF-α), reactive oxygen species (ROS), and inducible nitric oxide synthase (iNOS), which promote neuronal damage and exacerbate the severity of demyelinating lesions (Gerhauser et al., 2012; Luo et al., 2017; Shabani et al., 2020). In contrast, anti-inflammatory microglia (M2) are considered neuroprotective as they produce anti-inflammatory cytokines and neurotrophic factors such as brain neurotrophic derived factor (BDNF), insulin-like growth factor 1 (IGF-1), arginase-1 (Arg-1), and transforming growth factor β (TGF-β), which promote the expression of genes that are involved in the resolution of inflammation, and enhancing remyelination (Joseph and Venero, 2013; Hedl et al., 2016).

Although the MS etiology is still unknown, many studies report that MS is multifactorial and influenced by genetic and environmental factors that affect the nervous and immune systems (Dendrou et al., 2015; Qureshi et al., 2018). According to experimental and clinical findings, deficiency of vitamin D3 (Vit D3) is one of the environmental factors increasing the risk of developing and the prevalence of MS (Ascherio et al., 2010; Shoemaker and Mowry, 2018). As a result, MS physicians routinely prescribe oral supplements of Vit D3 to increase and maintain Vit D levels in MS patients (Dörr et al., 2020). Previous studies suggest that Vit D3 plays a pivotal role in immunomodulatory in MS (Chiuso-Minicucci et al., 2015; Mimura et al., 2016) and remyelination through stimulating the differentiation of oligodendrocyte progenitor cells (OPC; de la Fuente et al., 2015; Matías-Guíu et al., 2018). In addition, Wergeland et al. (2011) reported that administration of Vit D3 after 2 weeks of cuprizone (CPZ) withdrawal improved remyelination, and inhibited microglia/macrophage activation in the CPZ mouse model.

On the other hand, many oral disease-modifying therapies (DMTs) have been approved for MS treatment. Siponimod (Sipo) also named BAF312 is one of these drugs recently approved by the Food and Drug Administration (FDA) to treat patients with Secondary Progressive Multiple Sclerosis (SPMS; Behrangi et al., 2019b; Kremer et al., 2019). Sipo is a novel modulator of the sphingosine-1 receptor (S1P-R) that crosses the blood-brain barrier and selectively targets S1P1-R and S1P5-R; these receptors are involved in the migration of T cells into the CNS, microgliosis, astrogliosis, modulation of oligodendrocytes, and cell survival (Bigaud et al., 2014; O’Sullivan et al., 2016). Many preclinical studies have demonstrated that the Sipo attenuates demyelination in different mice strains and MS models (Gentile et al., 2016; Tiwari-Woodruff et al., 2016). At the same time, studies in experimental MS revealed that the Sipo reduces inflammation and lymphocyte migration in the CNS through S1PR1, which is distributed on astrocytes and microglia; in addition, it plays a crucial role in remyelination *via* S1PR5 expressed on oligodendrocytes (Noda et al., 2013; Luchtman et al., 2016). Gentile et al. (2016) reported that the administration of Sipo ameliorated clinical disability in experimental autoimmune encephalomyelitis (EAE) mice and significantly decreased astrogliosis and microgliosis in addition to inhibited synaptic neurodegeneration. Other studies showed the ability of BAF312 in attenuation of demyelination *via* modulation of glial cell processes implicated in cell survival (O’Sullivan et al., 2016), and stimulate remyelination in the reaggregated spheroid cell culture model (Jackson et al., 2011).

Remyelination is considered a crucial strategy for treating demyelinating diseases such as MS. The process of remyelination is closely related to the restoration of neuronal function and the alleviation of clinical disability (Harlow et al., 2015; Plemel et al., 2017). Currently, there is no effective therapeutic remyelination therapy, and it is crucial to develop potential therapeutic remyelination strategies for people suffering from demyelinating diseases (Hartley et al., 2014; Olsen and Akirav, 2015). In the context of microglia polarization in CNS inflammation and myelin loss, new therapeutic strategies aim to alter the CNS environment from M1 phenotype microglia polarization and stimulate remyelination and repair to slow and delay the complications of MS (Zhang et al., 2021).

An animal model is a valuable tool to mimic clinical human MS and to study the effect of different therapeutic strategies (Zhen et al., 2017). Cuprizone (CPZ) is a copper chelator that causes demyelination in the rodent CNS, mainly in the corpus callosum (CC), by inducing apoptosis of oligodendrocytes and activation of microglia and astrocytes within demyelinating lesions with minimal infiltration of peripheral immune cells into the brain (Matsushima and Morell, 2001; Steelman et al., 2012). Numerous pathologic features of histopathology in the CPZ mouse model resemble those found in MS lesions type III (Lucchinetti et al., 2000), Thus, exposure to CPZ results in behavioral abnormalities, impairment of motor skills, and alteration of mood, as observed in clinical demyelinating diseases (McMahon et al., 2002). Therefore, CPZ is an excellent experimental model for the study of de- and remyelination and a suitable pharmacological model for the development of new promising drugs for nerve protection and/or remyelination (Gudi et al., 2014).

To our knowledge, no previous study has investigated the therapeutic effect of the combination of Vit D3 and Sipo in the CPZ mouse model. Therefore, the current study investigated the therapeutic effect of a combination of Vit D3 and Sipo on promoting remyelination and alleviating motor behavioral deficits at early and late remyelination stages in the CPZ-induced demyelination model, as well as the potential effect of their combination in modulating microglial polarization.

## 2. Material and Methods

### 2.1. Animals

A total of 85 male Swiss mice (SWR/J), aged 6–7 weeks and weighing 18–21 g, were obtained from the King Fahd Medical Research Center (KFMRC), King Abdul-Aziz University, Jeddah, Saudi Arabia. Male mice were used because there was no difference in gliosis and demyelination in male and female mice when utilized CPZ to induce demyelination (Taylor et al., 2010). The mice were housed in groups (5 mice/cage) in a controlled temperature (23 ± 2°C) and humidity (65%) with a 12-h light/dark cycle and free access to food and water. The study protocol was approved by the biomedical ethics committee at King Abdul-Aziz University (Approval No. 551-20) and animal experiments were performed according to the animal unit committee’s guidelines at KFMRC.

### 2.2. Drug preparations

CPZ diet [0.3% (W/W)] was prepared by mixing 0.3 g CPZ powder (C9012-25G; St. Louis, MO, USA) manually with 100 g of ground rodent chow for 1 min and forming it in the pellet form (Nedelcu et al., 2020) to induce acute demyelination. The CPZ was prepared every 2 days and changed daily. Carboxymethylcellulose sodium salt (CMC, 1%) was prepared weekly by dissolving 1 gm of CMC (21902-100G; St. Louis, MO, USA) in 100 ml of water at a constant temperature of 60°C; and was stored at 4°C (Lan et al., 2018). Liquid Vit D3 (Cholecalciferol, 200 IU/drop, Pharmalink, E.U. Spain) was suspended in 1% CMC (800 IU/kg) based on a previous study (Borowicz et al., 2015). The tablet of Siponimod^®^ (2 mg Sipo; Novartis AG, EU) was weighed and gound into a powder and suspended in 1% CMC (1.5 mg/kg; Hundehege et al., 2019). Vit D3 and Sipo suspensions were prepared daily and administered to each mouse by oral gavage (0.2 ml /mouse); gavage was used to mimic the drug’s typical daily intake in humans. The dose of each drug was adjusted weekly based on the mice’s weight.

### 2.3. Experimental design

The total study period was 9 weeks, designed in two stages (de- and re-myelination). Mice were distributed randomly into two groups at the demyelination stage (first 5 weeks), control (*n* = 20) and CPZ (*n* = 65) groups. Mice in the control group received a standard rodent chow with *ad libitum* access during all experiment stages, and they received 0.2 ml of CMC orally during the last 4 weeks of remyelination stages. Mice in the CPZ group were fed a chow mixed with 0.3% CPZ for 6 weeks to induce acute demyelination, followed by a standard rodent chow for the last 3 weeks (remyelination stages). At week 5, the CPZ group was re-divided into four groups (*n* = 14): (1) untreated (CPZ) mice were administered 0.2 ml of CMC orally; (2) CPZ+Vit D3, mice were administered Vit D3 orally at 800 IU/kg; (3) CPZ+Sipo, mice were administered Sipo (1.5 mg/kg) orally; and (4) CPZ+ (Vit D3+Sipo), mice were administered Vit D3 (800 IU/kg) and Sipo (1.5 mg/kg) orally. CPZ exposure and drug treatment overlapped by 1 week to ensure that the remyelination was caused by the therapies and not due to spontaneous withdrawal of CPZ (Thompson et al., 2018; Nystad et al., 2020). This plan allowed treatment to begin during the disease’s progression, thus more closely resembling a therapeutic situation.

### 2.4. Body weight

The body weight of mice was measured at the end of every week in all groups at the same time. The change in weight gain (%) was calculated using the following equation: Body weight (%) = [new weight (W1) − initial weight (W0)/initial weight (W0)] *100 (Alghamdi, 2022).

### 2.5. Behavior tests

**Figure 1:**
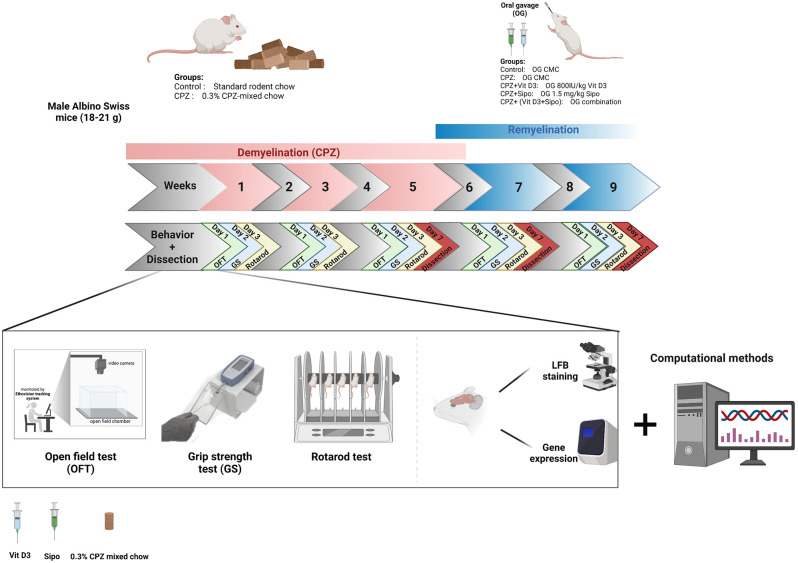
Timeline of the study and experimental design. CPZ, cuprizone; CMC, carboxymethyl cellulose; Vit D3, vitamin D3; Sipo, siponimod; OFT, open field test; GS, grip strength; LFB, Luxol fast blue. Created with https://biorender.com.

Behavior tests were performed every 2 weeks on separate days throughout the 9 weeks of the study in an isolated behavioral lab at room temperature (21°C–23°C) and under standard lighting conditions. Before testing, animals were habituated to their environment for 30 min on test days. The tests were carried out in the order shown in Figure 1.

#### 2.5.1. Open field test (OFT)

The Open Field is a simple test for evaluating locomotor function and exploratory movement in rodents around an open arena (Belzung and Griebel, 2001). In this study, the effect of CPZ and treatments during de- and re-myelination stages respectively were assessed on TDM (total distance moved) (cm) and velocity (cm/s). Each mouse was placed in the middle of the arena (a 45 × 45 cm square) and was allowed to move freely during the test and explore the surroundings for 3 min. The movement of the mice was tracked and the data was recorded by a video tracking program EthoVision XT8A system (Noldus Information Technology, Wageningen, The Netherlands) placed above the arena.

#### 2.5.2. Grip strength test (GS)

The grip strength test is a simple, non-invasive method designed for evaluating neuromuscular function by determining the maximal peak force generated by rodents when the operator pulls it out of a specific grid. In this study, the effect of CPZ and treatments during de- and re-myelination stages, respectively on the fore and hind limbs’ grip strength of each mouse was measured using a force gauge (Columbus Instruments, Columbus, OHIO 43204, USA). In brief, each mouse was placed carefully on the grip plate; after being held with all limbs, the mouse was pushed backward with its tail until it released the bar. The force (g) exerted on the grid when the grip was released was recorded on a digital force transducer and measured as the peak tension. Each mouse made three trials on each test day with a 30-min interval between trials, and the gauge was reset to 0 g between trials. The mean values (g) were calculated and normalized to body weight using the following formula: Normalized strength = (Force/body weight for each mouse) (Han et al., 2020; Ren et al., 2020).

#### 2.5.3. Rotarod test

The Rotarod test is a traditional behavioral test used to assess rodent motor-related abilities, such as coordination, balance, and fatigue (Dunham and Miya, 1957). In this experiment, the effect of CPZ and treatments on motor coordination and balance during de- and re- myelination, respectively were evaluated using a Rotarod apparatus (BR1001, B.S Technolab INC., Seoul, South Korea). Briefly, each mouse was placed on the rotating rod at 4 rpm and accelerated gradually to 40 rpm for 300 s; if a mouse fell off was stopped and the time was recorded. Three trials were performed on each test day per mouse (with a 1-h rest-pause between each trial), and the average latency to fall was recorded (Sen et al., 2020). The “latency to fall” is utilized as a quantitative endpoint to assess motor function.

#### 2.6. Brain tissue preparation

At the end of weeks 5, 7, and 9, mice were anesthetized with isoflurane and sacrificed. Brain tissues were collected, rinsed immediately with normal saline, and dissected into two hemispheres, left and right. The left halves were directly immersed into 10% formalin for histochemical examination [Luxol fast blue (LFB)]. The right halves were immersed in RNAprotect Tissue Reagent (Qiagen, Cat. No. 76104) for gene expression analysis. Tissue preparation and paraffin embedding were performed after a one-day fixation in formaldehyde. Serial sagittal sections were prepared by 5 μm thickness and stained with LFB.

#### 2.6.1. Luxol fast blue (LFB) staining

De- and re-myelination were detected histochemically in CC by staining brain tissues with LFB Stain (Klüver and Barrera, 1953) according to the manufacture’s protocol. After deparaffinization and rehydration, brain sections were incubated for 2 h in LFB solution (0.1%) at 60°C. LFB staining reveals the level of de and re-myelination through color variations; demyelinated areas appear in light blue, whereas myelinated areas show dark blue color. A light microscope (Olympus BX51) was used to examine sections, and images were taken with a digital camera (Olympus).

### 2.7. Gene interaction network

Gene-gene interactions of a network implicated in M1/M2 microglia phenotypes and myelin basic protein (MBP) myelination genes that interact with receptors of Vit D3, Sipo, and genes biomarkers related were generated using the GeneMANIA web tool^1^ (11 Jun 2022).

### 2.8 Quantitative real-time PCR (qRT-PCR)

Expression levels of the iNOS, IL-1β, IGF-1, and MBP in CC of mice were measured using qRT-PCR analysis. Total RNA was extracted from CC tissue using RNAbler—Cells and Tissue Kit (RE95050) according to the manufacturer’s protocol. complementary DNA (cDNA) was synthesized from RNA using ImProm-II^TM^ Reverse Transcription System (Promega; A3800) according to the manufacturer’s protocol. qRT-PCR was performed using BioFACT^TM^ 2X Real-Time PCR Master Mix (For SYBR^®^ Green I; DQ383-40h) according to the manufacturer’s instructions. Glyceraldehyde-3-phosphate dehydrogenase (GAPDH) is the primer used as a housekeeping gene (Kim et al., 2011). The double delta CT (2^−ΔΔct^) method was used to assess and normalized the mRNA levels. The primers used for real-time PCR are shown in Table 1.

**Table 1:** PCR primers sequences.

**Gene**	**Forward primer**	**Reverse primer**
GAPDH	ACCCAGAAGACTGTGGATGG	CACATTGGGGGTAGGAACAC
iNOS	CAAGCACCTTGGAAGAGGAG	AAGGCCAAACACAGCATACC
IL-1β	GCCACCTTTTGACAGTGATGAG	GACAGCCCAGGTCAAAGGTT
IGF-1	GGCATT GTGGATGAGTGTTG	TCTCCTTTGCAGCTTCGTTT
MBP	CAC ACA CGA GAACTA CCC A	GGT GTT CGA GGT GTC ACA A

GAPDH, Glyceraldehyde-3-phosphate dehydrogenase; iNOS, inducible nitric oxide synthase; IL-1b, interleukin 1b; IGF-1, insulin-like growth factor 1; MBP, myelin basic protein.

### 2.9 Molecular docking

The three-dimensional (3D)X -ray crystal structure of iNOS was retrieved from Protein Data Bank (PDB ID: 3E65, 2.00 Å; Garcin et al., 2008), which is bound with HEM and H4B as co-factors for function (Morris et al., 2009). Structures of ligands; Vit D3, Sipo, and L-Arginine as a standard ligand (CID: 5280795, 44599207, and 6322, respectively) were retrieved from the NCBI PubChem database. Molecular docking of these compounds on iNOS was done using AutoDock 4.2.6 and by selecting chain A. Then, by removing water molecules, they added hydrogen polar and charge. The ligand-protein interactions were calculated as the binding energy in kcal/mol and optimal interactions were chosen according to the best binding pose of various configurations with the lowest binding energy (kcal/mol) and a root mean square deviation (RMSD) of less than 2A. The PyMol (PyMOL Molecular Graphics System, Version 2.0, Schrödinger, LLC) and LigPlot+ v.1.4 were used to visualize these interactions.

### 2.10. Statistical analysis

All data were statistically analyzed using GraphPad Prism 9.2.0 and expressed as the mean standard error of the mean (SEM). Different tests conducted comparisons between groups; a t-test was conducted to analyze the percentage of myelination area and genes’ relative expression fold during the demyelination stage. One-way ANOVA followed by *post-hoc* Tukey’s test was conducted to analyze the percentage of myelination area and genes’ relative expression fold during remyelination stages. Two-way ANOVA followed by Šídák’s test was conducted to analyze body weight change%, TDM, velocity, grip force, and latency to fall in rotarod during the demyelination stage. Two-way ANOVA followed by Tukey’s test was conducted to analyze body weight change%, TDM, velocity, grip force, and latency to fall in rotarod during remyelination stages. If the *p-value < 0.05*, the differences between groups were considered statistically significant.

## 3. Results

### 3.1. Effects of Vit D3, Sipo, and their combination on body weight

**Figure 2:**
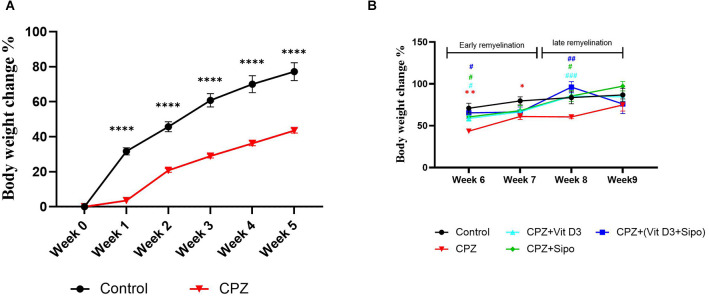
Body weight changes (%) of mice through experiment weeks. **(A)** Effects of CPZ on body weight changes (%) during the demyelination stage. **(B)** Effects of Vit D3, Sipo, and their combination on body weight changes (%) during remyelination stages. Data are presented as the mean ± SEM; two-way ANOVA followed by Šídák’s multiple comparisons test was used **(A)**, and two-way ANOVA followed by Tukey’s multiple comparisons test was used **(B)**. CPZ, cuprizone; Vit D3, vitamin D3; Sipo, siponimod. (*) Indicates a significant difference vs. the control group; (^#^) indicates a significant difference vs. CPZ group. *p < 0.05, **p < 0.01, ****p < 0.0001, ^#^*p* < 0.05, ^##^*p* < 0.01, and ^###^*p* < 0.001.

During 5 weeks of demyelination, two-way ANOVA repeated-measures exhibited a significant effect on weeks × groups [*F*_(5,415)_ = 47.85, *p* < 0.0001], weeks [*F*_(1.920,159.4)_ = 597.7, *p* < 0.0001], and groups [*F*_(1,83)_ = 132.3, *p* < 0.0001]. *Post-hoc* Šídák’s test showed that the CPZ reduced the weight gain % compared to the control group (*p* < 0.0001, Figure 2A).

On the other hand, Two-way ANOVA repeated measures exhibited a significant effect of weeks [*F*_(2.394,103.7)_ = 23.37, *p* < 0.0001] and groups [*F*_(4,70)_ = 4.687, *p* = 0.0021]. *Post-hoc* Tukey’s test revealed that CPZ significantly reduced the percentage of weight gain compared to the control group at the early remyelination stage in weeks 6 (*p* = 0.0031) and 7 (*p* = 0.0343) but not at the late remyelination stage in weeks 8 (*p* = 0.1094) and 9 (*p* = 0.7984). In contrast, Vit D3, Sipo, and their combination mediated the decrease in weight gain % compared with the CPZ group at the early stage in week 6 (*p* = 0.0118, *p* = 0.0285, *p* = 0.0357, respectively) and late stage in week 8 (*p* 0.0004, *p* = 0.0458, *p* = 0.0066, respectively). However, no significant change was observed in the percentage of weight gain at the early stage in week 7 in Vit D3 (*p* = 0.7147), Sipo (*p* = 0.8565), and Vit D3+ Sipo (*p* = 0.9365) and late stage in week 9 (*p* = 0.7517, *p* = 0.1708, *p* = >0.9999, respectively) compared to CPZ group (Figure 2B).

### 3.2. Effects of Vit D3, Sipo, and their combination on behavior functions

#### 3.2.1. Locomotor activity

**Figure 3:**
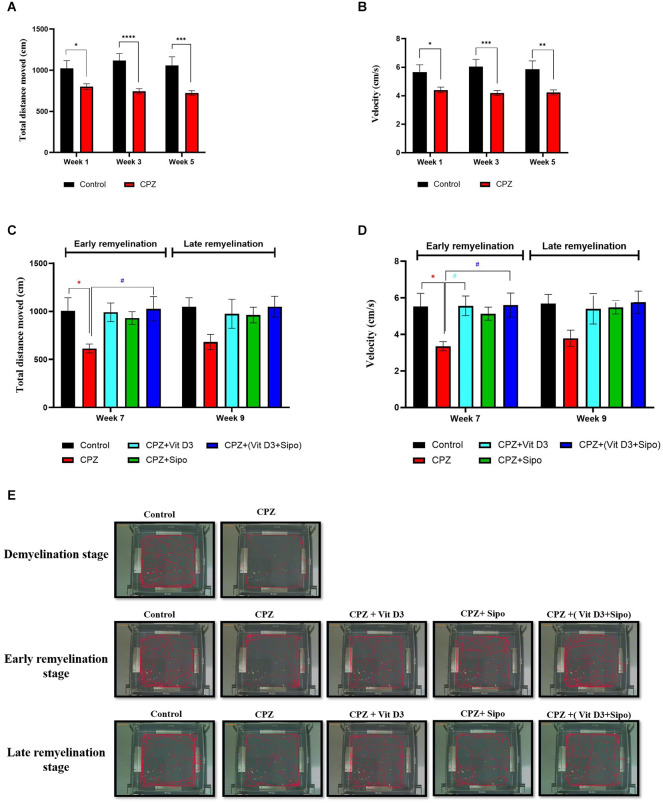
Locomotor activity. **(A,B)** Effects of the CPZ on the TDM and velocity in the OFT during the demyelination stage, respectively. **(C,D)** Effects of Vit D3, Sipo, and their combination on the TDM and velocity in OFT during (early and late) remyelination stages, respectively. Data are presented as the mean ± SEM; two-way ANOVA followed by Šídák’s multiple comparisons test was used **(A,B)**, and two-way ANOVA followed by Tukey’s multiple comparisons test was used **(C,D)**. **(E)** Representative images displaying typical examples of locomotor activity in an OFT in all groups during the different study stages. CPZ, cuprizone; Vit D3, vitamin D3; Sipo, siponimod. (*) Indicates a significant difference vs. the control group; (^#^) indicates a significant difference vs. the CPZ group. *p < 0.05, **p < 0.01, ***p < 0.001, ****p < 0.0001, and ^#^* p* < 0.05.

Two-way ANOVA repeated-measures exhibited a significant difference in the value TDM between groups [*F*_(1,219)_ = 44.2, *p* < 0.0001]. *Post-hoc* Šídák’s test showed that feeding with 0.3% CPZ caused a highly significant decrease in the TDM in week 1 (*p* = 0.0189), week 3 (*p* < 0.0001), and week 5 (*p* = 0.0001) compared with the control group during the demyelination stage (Figure 3A). Regarding the velocity, there was a significant difference in value among groups [*F*_(1,219)_ = 35.24, *p* < 0.0001]. *Post-hoc* Šídák’s test showed a significant decrease in the velocity in the CPZ group compared to the control group during the weeks of the demyelination stage week 1 (*p* = 0.0220), week 3 (*p* = 0.0002), and week 5 (*p* = 0.0015; Figure 3B).

At the remyelination stages, there was a significant difference in TDM value between groups [*F*_(4,75)_ = 4.670, *p* = 0.0020]. The value of TDM was significantly decreased in the CPZ (untreated) group compared with the control group (*p* = 0.0386) at the end of the early remyelination stage. While no significant change was observed between them at the late remyelination stage (*p* = 0.1671). In contrast, mice treated with Vit D3+Sipo exhibited increased TDM at the early stage compared with the CPZ group (*p* = 0.0256). However, no significant difference was seen at the late remyelination stage (*p* = 0.1637). Moreover, other treated groups showed no significant differences at early and late remyelination stages compared to the CPZ group, including CPZ+Vit D3 (*p* = 0.0519 and *p* = 0.3724, respectively) and CPZ+ Sipo (*p* = 0.1449 and *p* = 0.4165, respectively; Figure 3C).

There was a significant difference in velocity among groups at remyelination stages [*F*_(4,75)_ = 4.813, *p* = 0.0016]. The CPZ (untreated) group exhibited a decrease in velocity than the control group (*p* = 0.0315) at the early remyelination stage. However, no significant difference was seen at the late remyelination stage (*p* = 0.2022). On the other hand, mice treated with Vit D3 and Vit D3+Sipo significantly increased the velocity at the early stage compared with the CPZ group (*p* = 0.0270 and *p* = 0.0235, respectively), and no significant changes were observed at the late stage (*p* = 0.3543 and *p* = 0.1696, respectively). Moreover, no significant differences showed between CPZ+Sipo and CPZ groups at early and late remyelination stages (*p* = 0.1158 and *p* = 0.3048, respectively; Figure 3D). The EthoVision Tracking system calculated the data automatically as shown in Figure 3E.

#### 3.2.2. Grip strength

**Figure 4:**
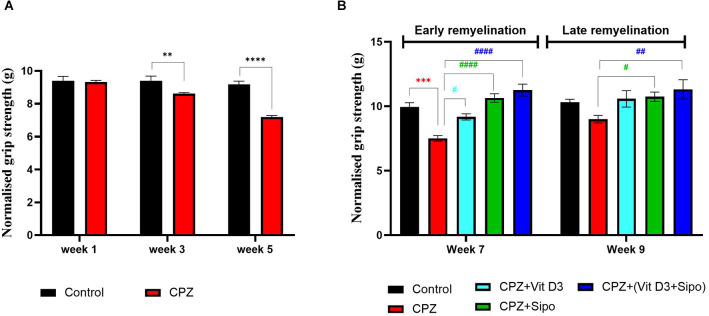
Grip strength test. **(A)** Effects of CPZ on the grip strength during demyelination stage. **(B)** Effects of different treatments on the mice grip strength during (early and late) remyelination stages. Data are represented as the mean ± SEM, and two-way ANOVA was used followed by Šídák’s and Tukey’s multiple comparisons tests **(A,B)** were used, respectively. CPZ, cuprizone; Vit D3, vitamin D3; Sipo: siponimod. (*) Indicates a significant difference vs. the control group; (^#^) indicates a significant difference vs. CPZ group. **p < 0.01, ***p < 0.001, ****p < 0.0001, ^#^*p* < 0.05, ^##^*p* < 0.01 and ^####^*p* < 0.0001.

Two-way ANOVA repeated-measures exhibited a significant difference in grip strength between weeks × groups [*F*_(2,219)_ = 18.73, *p* < 0.0001], weeks [*F*_(2,219)_ = 29.49, *p* < 0.0001], and groups [*F*_(1, 219)_ = 54.92, *p* < 0.0001]. *Post-hoc* Šídák’s test showed a highly significant decrease in the grip force in CPZ mice compared with control mice in weeks 3 (*p* = 0.0015) and 5 (*p* < 0.0001) during the demyelination stage (Figure 4A).

At the remyelination stages, there was a significant difference in grip strength between groups [*F*_(4,75)_ = 16.14, *p* < 0.0001] and weeks [*F*_(1,75)_ = 7.230, *p* = 0.0088]. Two-way ANOVA with Tukey’s test showed that grip force was significantly decreased in the CPZ (untreated) group compared with the control group at end of the early remyelination stage (*p* < 0.0001), but no significant difference was seen at the late remyelination stage (*p* = 0.2088). In contrast, mice treated with Vit D3, Sipo, and their combination exhibited increased grip strength at the early remyelination stage compared with the CPZ group (*p* = 0.0146, *p* < 0.0001, *p* < 0.0001, respectively). While, at the late remyelination stage, Sipo and Vit D3+Sipo showed significantly increased grip strength (*p* = 0.0462, *p* = 0.0031, respectively) compared with the CPZ group, but no significant change was seen in Vit D3 (*p* = 0.0880; Figure 4B).

#### 3.2.3. Motor coordination

**Figure 5:**
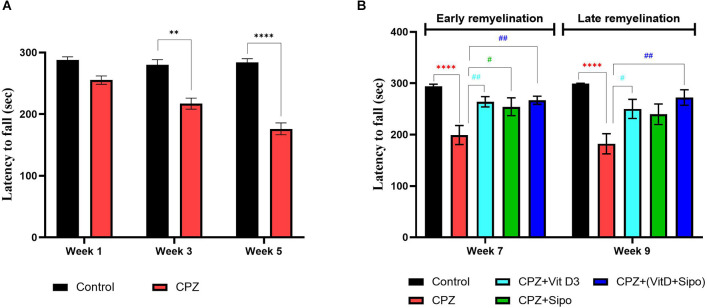
Rotarod test. **(A)** Effects of CPZ on the motor coordination and balance in the rotarod test during the demyelination stage. **(B)** Effects of different treatments on the motor coordination and balance in the rotarod test during early and late remyelination stages. Data are represented as the mean ± SEM, and two-way ANOVA was used followed by Šídák’s and Tukey’s multiple comparisons tests **(A,B)** were used, respectively. CPZ, cuprizone; Vit D3, vitamin D3; Sipo, siponimod. (*) Indicates a significant difference vs. the control group; (^#^) indicates a significant difference vs. CPZ group. **p < 0.01, ****p < 0.0001, ^#^*p* < 0.05, and ^##^*p* < 0.01.

Two-way ANOVA repeated-measures exhibited a significant difference in motor performance between weeks × groups [*F*_(2,219)_ = 4.747, *p* = 0.0096], weeks [*F*_(2,219)_ = 5.739, *p* = 0.0037], and groups [*F*_(1,219)_ = 45.86, *p* < 0.0001]. *Post-hoc* Šídák’s test showed a highly significant reduction in motor performance in the CPZ mice group compared with the control group in weeks 3 (*p* < 0.0001) and 5 (*p* < 0.0001) during the demyelination stage (Figure 5A).

At the remyelination stages, there was a significant variance in motor coordination among groups [*F*_(4,75)_ = 14.46, *p* < 0.0001]. Two-way ANOVA with Tukey’s test showed that motor performance was a highly significant decrease in the CPZ (untreated) group compared with the control group at end of the early (*p* < 0.0001) and late remyelination stages (*p* < 0.0001). In contrast, mice treated with Vit D3, Sipo, and their combination exhibited increased motor performance at the early remyelination stage (*p* = 0.0076, *p* = 0.0332, and *p* = 0.0045, respectively) compared with the CPZ group. However, at the late remyelination stage, Vit D3 and Vit D3+Sipo exhibited increased motor performance than the CPZ group (*p* = 0.0258 and *p* = 0.0012). While no significant change was observed in Sipo mice compared with the CPZ group (*p* = 0.0853; Figure 5B).

### 3.3. Effects of Vit D3, Sipo, and their combination on the histopathological changes of CC

**Figure 6:**
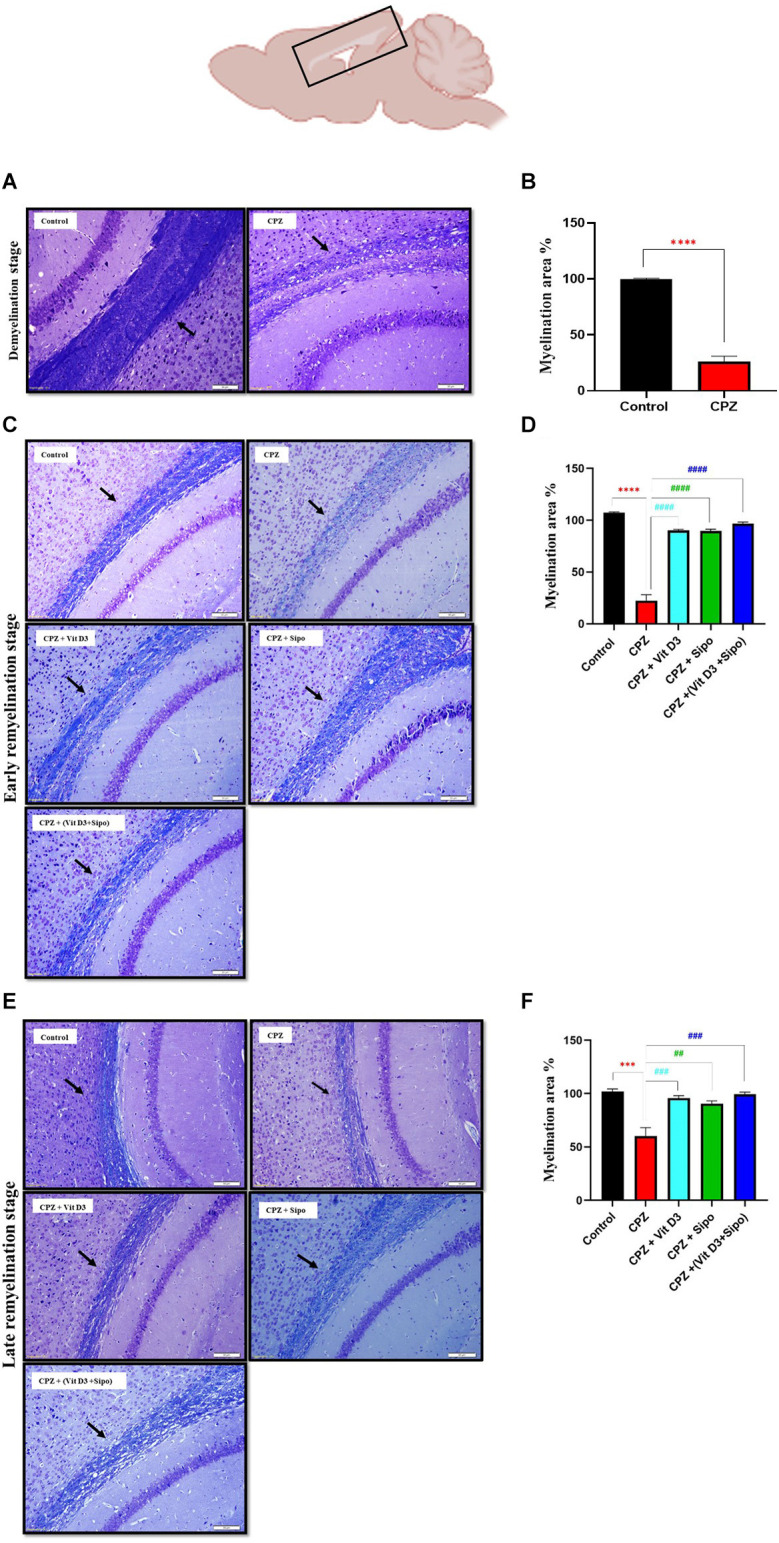
Evaluation of myelin content in sagittal sections of the CC using LFB staining and quantitative analysis of myelination area during de- and re-myelination stages. **(A)** Representative image of the CC using LFB staining during the demyelination stage in the control and CPZ group. **(B)** Percentage of myelination area in CC during the demyelination stage. **(C,E)** Representative images of LFB CC at the early and late remyelination stage in control and all treated groups, respectively. **(D,F)** Percentage of myelination area in CC during the early and late remyelination stage, respectively. CPZ administration for 5 weeks caused severe demyelination in the CC compared with the control mice. In contrast, different treatment groups ameliorated myelin injury and enhanced myelination in CC during remyelination stages. Data for Imag j are represented as the mean ± SEM; the t-test was used in **(B)**, and one-way ANOVA followed by the Tukey’s multiple comparisons test was used in **(D,F)**. Scale bars: 50 μm. CPZ, cuprizone; Vit D3, vitamin D3; Sipo, siponimod. (*) Indicates a significant difference vs. the control group; (^#^) indicates a significant difference vs. CPZ group. ***p < 0.001, ****p < 0.0001, ^##^*p* < 0.01, ^###^*p* < 0.001, and ^####^*p* < 0.0001.

De- and re-myelination were evaluated by histological examination of LFB-stain, which colors the lipid-rich myelin blue in the CC in brain sections. This region is consistently targeted by CPZ administration (Groebe et al., 2009). At the end of the demyelination stage, the LFB stains of the control CC tissue showed intact myelin sheath (complete myelination), which was demonstrated by the intense blue staining. While almost complete demyelination (much low level of myeline staining) was observed in the CPZ group compared with the control group, as shown in Figure 6A. Quantitative analysis of the myelinated area in the CC exhibited that the percentage of myelin area in the CPZ group decreased significantly (26.24%, *p* = 0.0001) compared with the control group (99.52%; Figure 6B).

At the remyelination stages, the sections from CPZ (untreated group) showed slight amelioration in myelin sheath in CC but still had a much lower level of staining compared with the control group. In contrast, the sections from mice treated with Vit D3, Sipo, and Vit D3+Sipo exhibited more amelioration in myelination in CC than in CPZ (untreated group), revealed by an intense blue color staining in the CC of the treated groups than CPZ group, as shown in Figures 6C,E. Moreover, quantitative analysis of the myelinated area in CC revealed that the percentage of myelin area in the CPZ group decreased significantly at early and late remyelination stages (22.34%, *p* < 0.0001 and 60.21%, *p* = 0.0001, respectively) compared with the control group (99.99%). At the same time, the percentage of myelin area in the treated groups significantly increased at early (CPZ+Vit D3 = 90.23%, *p* < 0.0001), (CPZ+Sipo = 89.72%, *p* < 0.0001), and (CPZ+(Vit D3+Sipo) = 96.63%, *p* < 0.0001) and late (CPZ+Vit D3 = 95.80%, *P* = 0.0008), (CPZ+Sipo = 90.54%, *p* = 0.0026), and (CPZ*+*(Vit D3+Sipo) = 99.45%, *p* = 0.0004) remyelination stages compared with the CPZ group (Figures 6D,F).

### 3.4. Network interaction

**Figure 7:**
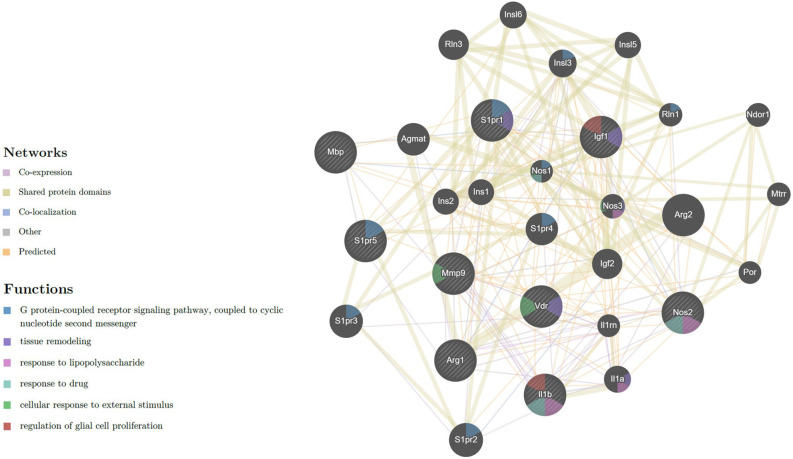
Network of expressed genes that related to M1/M2 microglia phenotype and myelination with receptors of Vit D3 and Sipo. VDR, MBP, S1PR5, S1PR1, MMP-9, Arg-1, IGF-1, iNOS, IL-1β, showing 20 related genes and 239 total links. VDR, vitamin D (1,25-dihydroxyvitamin D3) receptor; MBP, myelin basic protein; S1PR5, sphingosine-1-phosphate receptor 5; S1PR1, sphingosine-1-phosphate receptor 1; MMP9, matrix metallopeptidase 9; Arg1, arginase; IGF1, insulin-like growth factor 1; iNOS, inducible nitric oxide synthase; IL-1β, interleukin 1 beta; Arg2, arginase type II; S1PR2, sphingosine-1-phosphate receptor 2; S1PR3, sphingosine-1-phosphate receptor 3; Agmat, agmatine ureohydrolase; S1PR4, sphingosine-1-phosphate receptor 4; Rln3, relaxin 3; IGF-2, insulin-like growth factor 2; Insl3, insulin-like 3; Il1a, interleukin 1 alpha; Insl6, insulin-like 6; Insl5, insulin-like 5; Ins2, insulin II; Ins1, insulin I; Nos3, nitric oxide synthase 3, endothelial cell, Ndor1, NADPH dependent diflavin oxidoreductase 1; Mtrr, 5-methyltetrahydrofolate-homocysteine methyltransferase reductase; Rln1, relaxin 1; Il1rn, interleukin 1 receptor antagonist; Por, P450 (cytochrome) oxidoreductase; Nos1, nitric oxide synthase 1, neuronal. The nature of the identified interaction was indicated by the color of the line as seen in figure.

Network interaction between the nine expressed genes of the M1/M2 microglia phenotype and myelination, with receptors of Vit D3 and Sipo is displayed in (Figure 7), showing 20 related genes and 239 total links related to the activated microglia and remyelination. Ultimately, in this study, four genes (iNOS, IL-1β, IGF-1, and MBP) were selected as crucial genes to exhibit M1/M2 microglia markers and myelination gene.

### 3.5. Effects of Vit D3, Sipo, and their combination on genes expression level in the CC

#### 3.5.1. iNOS

**Figure 8:**
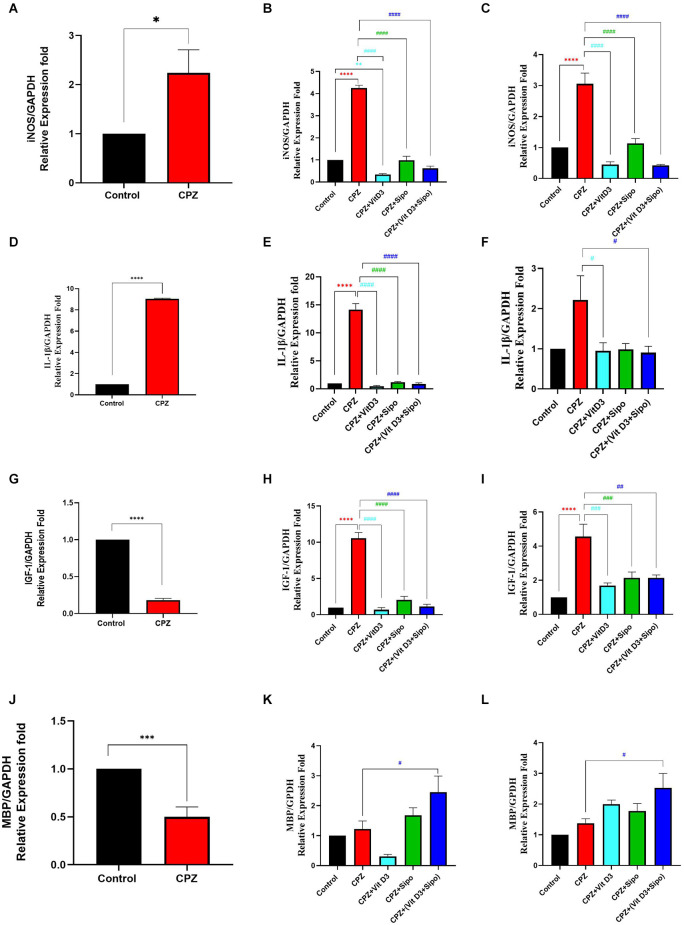
mRNA expression level of iNOS, IL-1β, IGF-1, and MBP in the CC. **(A,D,G,J)** Effects of CPZ on relative expression level of iNOS, IL-1β, IGF-1, and MBP in the CC of mice during demyelination stage, respectively. **(B,C,E,F,H,I,K,L)** Effects of Vit D3 and Sipo on relative expression level of iNOS, IL-1β, IGF-1, and MBP in the CC of mice during early and late remyelination stages, respectively. Data are represented as means ± SEM and t-test was used in **(A,D,G,J)** and One-way ANOVA followed by Tukey’s test was used in **(B,C,E,F,H,I,K,L)**. iNOS, inducible nitric oxide synthase; IL-1β, interleukin 1 beta; IGF-1, insulin-like growth factor 1; MBP, myelin basic protein; GAPDH, glyceraldehyde-3-phosphate dehydrogenase; CPZ, cuprizone; Vit D3, vitamin D3; Sipo, siponimod. (*) Indicates a significant difference vs. the control group; (^#^) indicates a significant difference vs. CPZ group. *p < 0.05, **p < 0.01, ***p < 0.001, ****p < 0.0001, ^#^*p* < 0.05, ^##^*p* < 0.01, ^###^*p* < 0, 001, and ^####^*p* < 0.0001.

During the demyelination stage, iNOS levels were significantly increased in the CC tissues in the CPZ group compared to the control group (*p* = 0.0254), as shown in Figure 8A. During the early and late remyelination stages, there was a significant difference in iNOS expression level [*F*_(4,25)_ = 217.1, *P* < 0.0001] and [*F*_(4,25)_ = 37.61, *P* < 0.0001], respectively. The expression level of iNOS was still significantly high in the CPZ group compared to the control group in the early and late remyelination stages (*p* < 0.0001, Figures 8B,C). In contrast, a significant decrease in iNOS expression level was observed in all treated groups, including CPZ+Vit D3 (*p* < 0.0001), CPZ+Sipo (*p* < 0.0001), and CPZ+ (Vit D3+Sipo; *p* < 0.0001) compared to CPZ group at the early and late remyelination stages (Figures 8B,C).

#### 3.5.2. IL-1β

During the demyelination stage, the expression level of IL-1β was significantly increased in the CC tissues of the CPZ group compared with the control group (*p* < 0.0001), as shown in Figure 8D. During the early remyelination stage, there was a significant difference in IL-1β expression level [*F*_(4,25)_ = 147.9, *P* < 0.0001]. *Post-hoc* Tukey’s test showed that the IL-1β expression level was significantly increased in the CPZ group compared to the control group (*p* < 0.0001). In contrast, the treated groups, including CPZ+Vit D3 (*p* < 0.0001), CPZ+Sipo (*p* < 0.0001), and CPZ+(Vit D3+Sipo; *p* < 0.0001) exhibited a significant decrease in the level of IL-1β compared to the CPZ group (Figure 8E). On the other hand, during the late remyelination stage, there were significant differences in the level of IL-1β expression among groups [*F*_(4,25)_ = 3.553, *P* = 0.0199]. No significant change in the level of IL-1β expression was seen in the CPZ group compared to the control group (*p* = 0.0566). While CPZ+Vit D3 and CPZ+(Vit D3+Sipo) exhibited a significant decrease in IL-1β level compared to the CPZ group (*p* = 0.0441 and *p* = 0.0343, respectively). However, the CPZ+Sipo group showed no significant change in IL-1β expression compared to the CPZ group (*p* = 0.0527; Figure 8F).

#### 3.5.3. IGF-1

During the demyelination stage, there was a significant decrease in the level of IGF-1 expression in CC tissues of the CPZ group compared to the control group (*p* < 0.0001, Figure 8G). During the early and late remyelination stages, there was a significant difference in IGF-1 expression level [*F*_(4,25)_ = 84.03, *P* < 0.0001] and [*F*_(4,25)_ = 12.89, *P* < 0.0001], respectively. *Post-hoc* Tukey’s test showed a significant increase in IGF-1 level in the CPZ group compared with the control group at the early and late remyelination stages (*p* < 0.0001, Figures 8H,I). In contrast, the treated groups exhibited a significant decrease in IGF-1 levels at both remyelination stages, including CPZ+Vit D3 (*p* < 0.0001), CPZ+Sipo (*p* < 0.0001 and *p* < 0.0010, respectively), and CPZ+(Vit D3+Sipo; *p* < 0.0001 and *p* < 0.0010, respectively) compared to the CPZ group (Figures 8H,I).

#### 3.5.4. MBP

During the demyelination stage, there was a significant decrease in the level of MBP in the CC in the CPZ group compared with the control group (*p* = 0.0006, Figure 8J). During the early and late remyelination stages, there was a significant difference in the expression level of MBP [*F*_(4,25)_ = 7.418, *P* = 0.0004] and [*F*_(4,25)_ = 5.358, *P* = 0.0029], respectively. No significant change was observed in the expression level of MBP in the CPZ group at the early and late remyelination stages compared to the control group (*p* = 0.9830 and *p* = 0.8252, respectively). On the other hand, the level of MBP was significantly increased in the CPZ+(Vit D3+Sipo) group at both remyelination stages compared to the CPZ group (*p* = 0.0463 and *p* = 0.0267, respectively). However, no significant change was observed in the CPZ+Vit D3 (*p* = 0.2119 and *p* = 0.4325) and CPZ+Sipo (*p* = 0.7994 and *p* = 0.8000) compared to the CPZ group at the early and late remyelination stages, respectively (Figures 8K,L).

### 3.6. Molecular docking analysis and prediction of potential binding site

**Figure 9:**
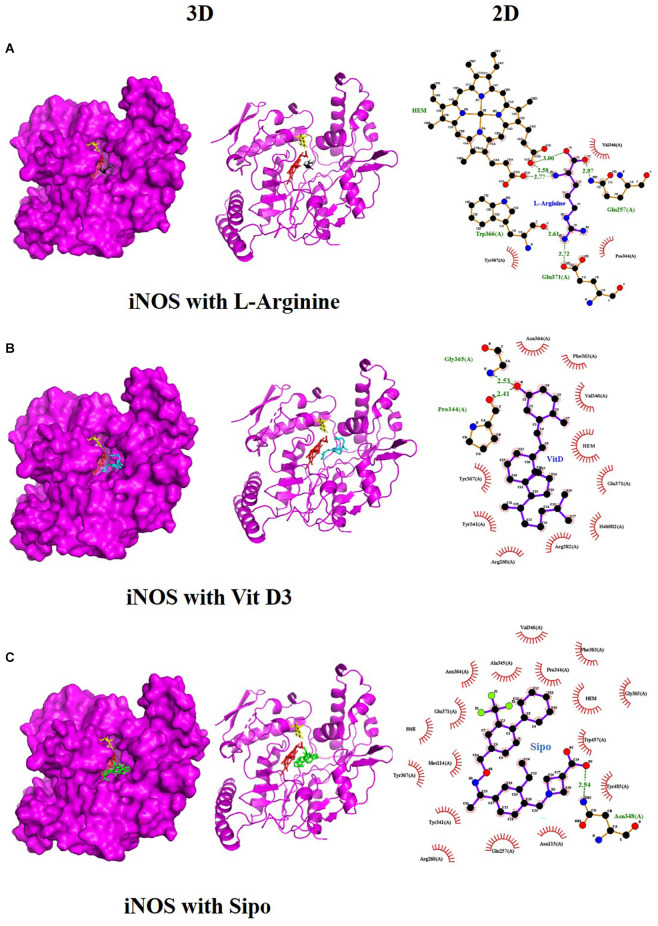
Interaction between iNOS and **(A)** L-Arginine, **(B)** Vit D3, and **(C)** Sipo at the binding site, represented as 3D and 2D, respectively. Green spoked arcs represent hydrogen bonds, and brick red spoked arcs represent hydrophobic interactions. iNOS, inducible nitric oxide synthase; Vit D3, vitamin D3; Sipo, siponimod.

We used molecular docking to predict the potential of Vit D3 and Sipo as inhibitors of iNOS and compared them with L-arginine as an agonist of it. Both drugs were shown to occupy the active site of iNOS, as illustrated in Figure 9. The protein-ligand interaction has a higher affinity when the binding energy is lower. The binding affinity of Vit D3 was observed to be high (−11.67 Kcal/mole), which formed two hydrogen bonds with Gly365 and Pro334 (2.53 and 2.41 Å), while the binding affinity of Sipo was −8.2 Kcal/mole *via* interaction with Asn348 (2.94 Å) as shown in Table 2. Besides, hydrophobic interaction surrounds Sipo more than Vit D3. In contrast, L-arginine was bound with a low affinity that was −7.14 Kcal/mole, though it formed three hydrogen bonds *via* Typ366, Gln257, and Glu371 residues.

**Table 2:** Molecular docking score for the Vit D3, Sipo, and L-Arginine with iNOS protein.

**PDB ID**	**3E65**		
**Ligand name**	**Vitamin D3**	**Siponimod**	L-Arginine*
**Ligand structure**	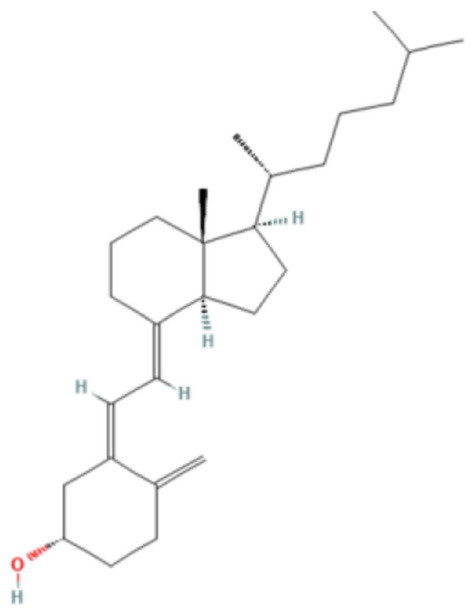	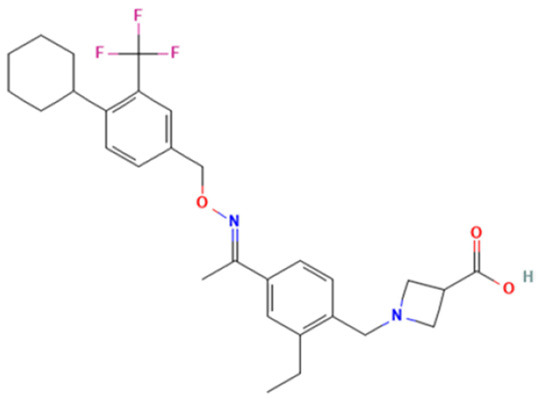	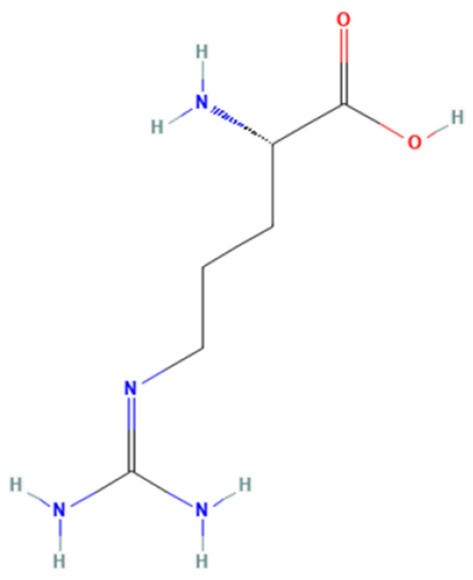
**Binding Energy (Kcal/mol)**	−11.67 Kcal/mole	−8.2 Kcal/mole	−7.14 Kcal/mole
**Hydrophobic interactions**	Sn364(A), Phe363(A), Val346(A), HEM, Glu371(A), H4b902(A), Arg382(A), Arg260(A), Tyr341(A), Tyr367(A)	Val346(A), Ala345(A), Pro344(A), Phe363(A), HEM, Gly365(A), Trp457(A), Tyr485(A), Asn115(A), Gln257(A), Arg260(A), Tyr341(A), Met114(A), Tyr367(A), H4B, Glu371(A), Asn364(A)	Val346(A), Pre344(A), Tyr367(A)
**Hydrogen bond**	Gly365(2.53Å) Pro334(2.41Å)	Asn348 (2.94Å)	Typ366 (2.61Å), Gln257 (2.79Å), Glu371(1.72Å)

*L-Arginine used as ligand reference.

## 4. Discussion

In general, Vit D3 supplementation shows promising results in reducing the progression of motor deterioration and promoting remyelination in various MS models (Shirazi et al., 2017; Nystad et al., 2018; Oveland et al., 2018; Hoepner et al., 2019). Thus, our objective was to evaluate the potential therapeutic effects of a combination of Vit D3 and Sipo treatment on enhancing remyelination and ameliorating motor defects during early and late remyelination stages in a CPZ mouse model, focusing on the effects of these treatments on modulating microglial activation. Our results indicated that administration of a combination of Vit D3 and Sipo improved remyelination by ameliorating behavioral deficits and histological changes in CC and modulating the expression of MBP and other genes related to M1 markers of microglia activation during remyelination stages.

In several experimental studies, loss of body weight after CPZ intoxication was a surrogate indicator of the desired demyelination activity of CPZ* in vivo* (Hiremath et al., 1998; Yu et al., 2017). In our study, it was found that the body weight of the CPZ mice group was significantly decreased compared with the control group, which is in line with previous experimental studies indicating that the body weight of the CPZ mouse model of MS was significantly lower than that of the control mice (Omotoso et al., 2018; He et al., 2019; Mojaverrostami et al., 2020; Wang J. et al., 2020). Approximately 80% of patients with MS have deficits in motor functions, such as difficulty maintaining balance, decreased walking speed, and impaired dexterity of the hands and feet (LaRocca, 2011; Pellegrino et al., 2018). In this context, we performed several behavioral tests, including the open-field test, grip strength test, and rotarod test, to assess motor function deficits in an animal model (Sen et al., 2020). Altogether, our results showed that CPZ administration for 5 weeks resulted in decreased locomotor activity, as indicated by the lowered TDM and velocity, a significant decrease in grip strength, and motor coordination in the CPZ mice group compared with the control group, which is consistent with previous findings (Soundarapandian et al., 2011; Faizi et al., 2016; Wang et al., 2016; Sanadgol et al., 2017; Elbaz et al., 2018; Kumar et al., 2018; Yu et al., 2018; Han et al., 2020; Wang S.-S. et al., 2020). The decreased motor coordination and induced behavioral deficits in the CPZ mouse model are due to the severe demyelination, activation of microglia and astrocytes, and increased oligodendrocyte apoptosis in the CC, as well as the decreased level of myelin protein as MBP and proteolipid protein (PLP; Mojaverrostami et al., 2020; Wang S.-S. et al., 2020; Zhang et al., 2021). We found severe demyelination in the CC after administration of CPZ for 5 weeks as assessed by LFB staining and decreased expression level of MBP compared to the control group; this finding confirmed our behavior results suggesting that the behavioral impairment in mice is paralleled with demyelination in the brain (Frid et al., 2015; Zendedel et al., 2016; Hochstrasser et al., 2017; Mojaverrostami et al., 2020). MBP is the main component of myelin in the CNS and a good marker for showing early remyelination processes (Lindner et al., 2008; Jahn et al., 2009). It plays the main role in myelin membrane compaction and stability through a precise stoichiometric balance between the basic MBP residues and the acidic headgroups of the lipid bilayer components and on copper ions that cause compaction of the MBP structure (Baran et al., 2010; Fulton et al., 2010). Reduced myelin stability could be related to changes in the levels of different myelin components, such as copper ions, MBP expression levels, or certain lipids and their oxidized products (Ferretti and Bacchetti, 2011). When demyelination occurs as a result of CPZ administration, MBP may either be rapidly degraded or combine with other myelin components due to the removal of copper ions, resulting in loss of myelin compaction (Baran et al., 2010). Moreover, we examined the relative expression levels of genes correlated to the microglia activation in the CC during different study stages, including iNOS, IL-1β, and IGF-1, and we found a highly significant increase in the expression of iNOS and IL -1β and a significant decrease in the expression of IGF-1 in CC the mouse model, which is consistent with previous findings that indicated the effect of CPZ on the activation of microglia and increasing the expression level of iNOS (Barati et al., 2019; Zhang et al., 2021) and IL-1β (Kim et al., 2018). iNOS enzyme in activated microglia releases NO (nitric oxide), which degrades the myelin sheath, kills oligodendrocytes, and damages axons (Sloka and Stefanelli, 2005). Moreover, IL-1β, a cytotoxic component of the inflammasome, caused oligodendrocyte damage *in vivo* and *in vitro*, stimulating microglia and astrocyte proliferation, and release several cytokines, including TNF-, IL-6, and NO, which contribute to the inflammatory response (Giulian et al., 1988; Takahashi et al., 2003). Many studies found a relationship between IL-1β and the severity of MS, whereby increased levels of IL-1β in MS patients’ cerebrospinal fluid were linked to the relapse-onset MS progression (Feakes et al., 2000; Niino et al., 2001; de Jong et al., 2020). IGF-1 is a growth factor that induces the proliferation and differentiation of oligodendrocytes progenitors* in vitro* (Glazebrook et al., 1998). Additionally, IGF-1 plays a vital role in the myelination process by activating type 1 receptors (IGF1R; Liu et al., 1993). Previous studies showed the increased level of IGF-1 in adult CNS demyelinating and remyelinating lesions to protect mature oligodendrocytes during the demyelination stage, hence stimulating a rapid recovery (Yao et al., 1995; Mason et al., 2000a, b).

At the remyelination stages of this CPZ group, our results exhibited that the body weight of the CPZ group was still a highly significant reduction during the early remyelination stage (weeks 6 and 7) compared to the control group. Whereas there was normal body weight gain during the late remyelination stage after 2 and 3 weeks of CPZ cessation (weeks 8 and 9, respectively). These results are consistent with previous findings reporting that spontaneous remyelination occurs after 9 days of CPZ withdrawal, and mice rapidly gain weight, similar to the vehicle group (Franco-Pons et al., 2007; Tagge et al., 2016; Zhen et al., 2017; Omotoso et al., 2018; Mojaverrostami et al., 2020). On the other hand, behavioral defects, elevated levels of iNOS, IL-1β, and IGF-1, and low intensity of LFB in CC continued after 1 week of CPZ withdrawal match with a previous study (Franco-Pons et al., 2007). In addition, one study showed that IL-1β in the CPZ model increased gradually from the first week of CPZ intoxication, then increased significantly at week 3, and remained at this level until week 6 (Mason et al., 2001); this explains the reason increase in the expression level of IL-1β during early remyelination stage. While, at a late stage of remyelination, all these outcomes improved as in the control group, except for rotarod performance and iNOS and IGF-1 levels, which remained unchanged. These results are consistent with previous findings (Chami et al., 2017; Wang J. et al., 2020; Yin et al., 2020) and confirm our study design, in which we examined our results at different time points during remyelination to avoid the effect of spontaneous remyelination by CPZ withdrawal on therapeutic intervention and to see whether the drugs can alter clinical symptoms shortly after disease onset, simulating treatment in humans. Overall, our results successfully established the CPZ-induced demyelination model in mice, and it is an excellent model for generating many essential features of MS disease and could be used to perform remyelination tests in this study.

Regarding the effect of Vit D3, Sipo, and their combination at remyelination stages, our results showed that the Vit D3 administration ameliorated the weight changes in the early (week 6) and late (week 8) remyelination stages compared with the CPZ group. This result is consistent with previous studies (Nystad et al., 2014; Gomez-Pinedo et al., 2020). In addition, we found that the administration of Sipo alone or in combination with Vit D3 also alleviated the weight changes at early and late remyelination stages (weeks 6 and 8, respectively) compared with the CPZ group. To our knowledge, no previous study has examined the treatment effect of Sipo and the combination of Vit D3+Sipo on behavioral deficits in the CPZ mouse model. Our results showed that treatment with Vit D3 improved behavioral deficits, as evidenced by increased velocity and improved grip strength at the end of the early remyelination stage and improved motor coordination in both remyelination stages compared with the CPZ group, consistent with previous findings (Camargo et al., 2018, 2020). In addition, we found that treatment with Sipo also improved grip strength and motor coordination at the end of the early and late remyelination stages compared with the CPZ group, whereas there was no significant effect on motor activity. Interestingly, the combination of Vit D3 and Sipo resulted in a significant improvement in all behavioral disorders; this was evidenced by improved locomotor activity at the early remyelination stage by increasing TDM and velocity compared to the CPZ group, as well as significantly improved grip strength and motor coordination at the early and late remyelination stages.

Interestingly, our results showed that treatment with Vit D3 or Sipo significantly improved remyelination in the CC at the end of the early and late remyelination stages at the histological level as assessed by LFB staining compared with the CPZ group. This result is consistent with previous studies reporting that Vit D3 administration significantly increased the remyelination rate in the middle of CC and the spinal cord in CPZ and EAE animal models, respectively (Goudarzvand et al., 2010; Wergeland et al., 2011; Nystad et al., 2014; De Oliveira et al., 2020), and the use of Sipo enhances remyelination in CPZ mouse models through interactions with innate CNS immune cells (Behrangi et al., 2019a). In contrast, no significant change in the mRNA expression level of the MBP gene was detected after treatment with Vit D3 or Sipo at the early and late remyelination stages compared with the CPZ group. One possible explanation for this discrepancy is the sensitivity of LFB to detect partial changes in myelin density in MS (Seewann et al., 2009). In addition, the highly significant decrease in the expression level of MBP in the Vit D3 group during the early remyelination stage could be attributed to no effect of Vit D3 after 2 weeks from its administration, since cholecalciferol requires some time to be enzymatically converted to calcitriol, a biologically active form of Vit D, to have an effect. Interestingly, we found that the combination of Vit D3 and Sipo treatment significantly improved remyelination in CC at the histological and transcriptional levels of MBP. One possible explanation for this result is that abundant expression of Vit D receptor (VDR) and S1PR5 on oligodendrocytes plays an important role in myelination and increases the expression of MBP and thus the stability of myelin membranes (Eyles et al., 2005; Mattes et al., 2010).

To confirm the positive effects of Vit D3 and Sipo (alone or in combination) on behavior defects and remyelination rate, the expression levels of iNOS, IL-1β, and IGF-1 secreted by microglia were identified by QRT-PCR. Our results showed that treatment with Vit D3 significantly decreased the expression levels of iNOS, IL-1β, and IGF-1 in CC at end of early and late remyelination stages compared with the CPZ group. These findings are consistent with previous studies reporting that treatment with Vit D3 significantly ameliorated neuroinflammation in the 1-methyl-4-phenyl-1,2,3,6-tetrahydropyridine (MPTP) animal model of Parkinson’s disease (PD) *via* shifting responses of microglia from pro-inflammatory (M1) to anti-inflammatory (Calvello et al., 2017). In addition, Cui et al. (2019) reported that the administration of calcitriol suppressed microglia M1 polarization while increasing M2 polarization in addition to reduced expression of pro-inflammatory cytokines in spontaneously hypertensive rats *via* induced the expression of VDR. On the other hand, we found that the administration of Sipo also significantly decreased the expression levels of iNOS, IL-1β, and IGF-1 in CC at the end of both remyelination stages compared to the CPZ group. This is in line with a previous finding reporting that treatment with (1 mg/kg) of fingolimod (FTY720) ameliorated injury to OLGs, axons, and myelin in the CC of the CPZ mouse model and attenuated microgliosis *via* decreased the IL-1β, IGF-1, and CCL2 expression levels in CC (Kim et al., 2011). Another study revealed that an S1P1/S1P5 modulator (ozanimod) ameliorated inflammatory glutamate-mediated excitotoxicity in an EAE animal model *via* the effect on microglia activation and decreased release of proinflammatory cytokines as TNF-α, IL-1β, IL-6, and iNOS (Musella et al., 2020). Interestingly, the combination of Vit D3 and Sipo exhibited a highly significant reduction in the expression levels of iNOS, IL-1β, and IGF-1 in CC at early and late remyelination stages.

Molecular docking was carried out to validate the action of Vit D3 and Sipo on iNOS to evaluate the therapeutic potential of the drug used in this study. A heme macrocycle and tetrahydrobiopterin (H4B) are involved in the activity of iNOS’ binding pocket, which has Glu371, Gln257, Tyr341, Arg260, Arg382, Thr277, Phe280, and Leu299r residues (Zhang et al., 2012). L-arginine is a substrate for iNOS, which also interacts with heme to produce NO (Garcin et al., 2008). Based on the computational interaction between Vit D3 and Sipo with iNOS, it was revealed that both are bound to the binding pocket of iNOS with the highest affinity for L-arginine and increased hydrophobic interaction in Sipo due to aromatic rings in its structure. Therefore, these bindings could block the binding site and thereby inhibit or reduce the activity of iNOS, which is one of the target steps for the treatment of inflammatory diseases (Zhang et al., 2012). In RT-PCR, we found that combining Vit D3 and Sipo inhibited iNOS activity, implying that shallow pockets of iNOS accommodated both medications and prevented L-arginine from binding. The process of molecular docking explains this.

It is worth mentioning that the widely distributed VDR and S1P receptors (S1PR1 and S1PR5) on neurons, oligodendrocytes, astrocytes, and microglia (Eyles et al., 2005; Novgorodov et al., 2007; Van Doorn et al., 2010; Groves et al., 2013; Smolders et al., 2013) could be responsible for the positive effects of the combination of Vit D3 and Sipo aforementioned as a synergy of their activities. In addition, Sipo, a SIP receptor modulator, crosses the Blood Brain Barrier (BBB) more readily than sphingosine-1-Phosphate (S1P) due to its size and lipophilic nature and prevents S1P from binding to S1P receptors. S1P influences various physiological and pathological processes, including migration, cytoskeleton remodeling, inflammation, proliferation, and apoptosis, *via* five G-protein-coupled receptors (S1P1-S1P5; O’Brien et al., 2009). In addition, Vit D3 has a protective effect in the MS models by decreasing S1P levels (Zhu et al., 2014). In this context, the combination of Vit D3 and Sipo could increase the binding of Sipo to its receptors and enhance its activity. Undoubtedly, there are still some limitations in this study, such as the lack of immunohistochemical examination of microglial activity and the number of astrocyte and oligodendrocyte cells. On the other hand, no study has yet investigated the effect of the combination of Vit D3 and Sipo on cognitive impairment. Therefore, we recommend that the effect of these combinations on cognitive impairment in CPZ-induced demyelination be investigated.

## 5. Conclusion

**Figure 10:**
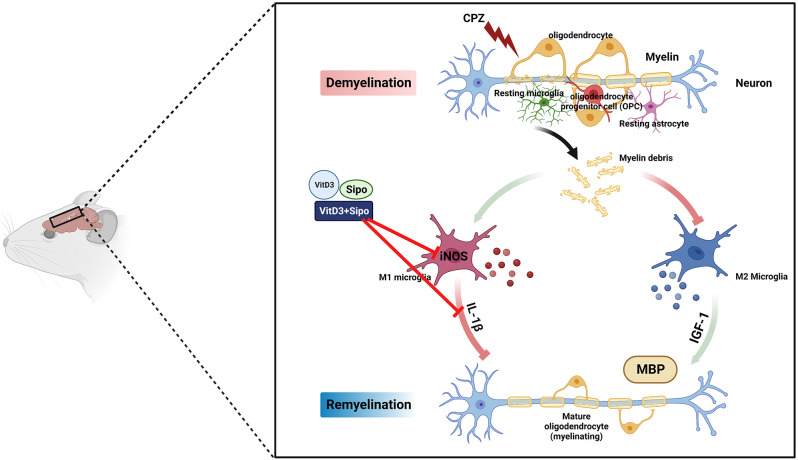
Schematic representation of the potential therapeutic effects of Vit D3, Sipo, and their combination on gene expression of M1 and M2 microglia markers (iNOS, IL-1β, IGF-1) and myelination (MBP). CPZ, cuprizone; IL-1β, interleukin 1 beta; iNOS, inducible nitric oxide synthase; IGF-1, insulin-like growth factor 1; MBP, myelin basic protein; Vit D3, vitamin D3; Sipo, siponimod. Red lines represent inhibitory effects. Created with BioRender.com.

Our results demonstrate for the first time the potential synergistic effects of Vit D3 and Sipo on enhanced remyelination in mice with CPZ-induced demyelination. Therapeutic effects of this combination were observed at several levels: improvement of behavioral deficits, reduction of M1-like microglia markers, and increased MBP expression, which promotes myelination (Figure 10). Therefore, combining these two treatments to treat MS patients may represent a novel strategy to accelerate remyelination, alleviate disease symptoms, and improve quality of life.

## Data Availability Statement

The original contributions presented in the study are included in the article, further inquiries can be directed to the corresponding authors.

## Ethics Statement

The animal study was reviewed and approved and the experimental procedures were approved by the biomedical ethics research committee (Approval No. 551-20) at King Abdulaziz University.

## Author Contributions

KA-O and UO conceived the concept and designed the study. KA-O prepared material, performed experiments, analyzed the data, and wrote the manuscript. BA assisted in the experiments and data analysis. All authors contributed to the article and approved the submitted version.

## Footnotes

^1^ https://genemania.org/
